# Associations between Psychological Distress, Perceived Social Support and Physical Activity Level in Spanish Adults with Depression

**DOI:** 10.3390/healthcare10091620

**Published:** 2022-08-25

**Authors:** Ángel Denche-Zamorano, Raquel Pastor-Cisneros, Jorge Carlos-Vivas, Juan Manuel Franco-García, Damián Pereira-Payo, Sabina Barrios-Fernandez, Jorge Rojo-Ramos, María Mendoza-Muñoz

**Affiliations:** 1 Promoting a Healthy Society Research Group (PheSo), Faculty of Sport Sciences, University of Extremadura, 10003 Caceres, Spain; 2 Social Impact and Innovation in Health (InHEALTH), University of Extremadura, 10003 Caceres, Spain; 3 Health, Economy, Motricity and Education (HEME) Research Group, Faculty of Sport Sciences, University of Extremadura, 10003 Caceres, Spain; 4 Research Group on Physical and Health Literacy and Health-Related Quality of Life (PHYQOL), University of Extremadura, 10003 Caceres, Spain

**Keywords:** physical activity, mental health, stress, psychology, health survey

## Abstract

Perceived social support (PSS) and physical activity (PA) could help to reduce psychological distress in people with depression. This study aims to analyse the associations between (a) mental health and its dimensions through the Goldberg General Health Questionnaire (GHQ-12), (b) the PA level (PAL), and c) the PSS in the Spanish adult population with psychological distress. This cross-sectional study is based on Spanish National Health Survey 2017 data, including 1670 adults with depression. A descriptive analysis was performed. Differences in medians between sexes were analysed using the Mann–Whitney U test. The Chi-square test was used to assess the independence between sex and PAL. The Kruskal–Wallis’ test was performed to analyse possible baseline differences between PAL and continuous variables derived from the GHQ-12. Finally, a correlation study was conducted between the generated variables and the GHQ-12 items, together with the PAL and the Duke-UNC-11, using Spearman’s rho correlation coefficients. Weak inverse correlations were found between the GHQ-12 and PAL (rho: −0.214); and PSS (r: −0.286). PAL and PSS showed weak inverse correlations with successful coping (rho: −0.216 and r: −0.265), self-esteem (rho: −0.209 and r: −0.283), and stress (rho: −0.130 and r: −0.232). Thus, higher PAL and SSP is associated with lower psychological distress.

## 1. Introduction

According to the World Health Organization (WHO), mental health is the state of well-being in which individuals can perform their abilities, cope with their daily stress and obligations, work productively, and contribute to their community [1]. Therefore, the lack of mental health can lead to mental disorders, mainly concerning two main diagnostic categories: depressive and anxiety disorders [2]. Depression is a syndrome involving a state of “dysphoric mood” characterised by feeling sad, hopeless, irritable, or losing interest and pleasure [3]. Moreover, depression is one of the most critical risk factors for suicide, the second leading cause of mortality among young adults in Spain [4], and is associated with increased morbidity and mortality [5,6], non-communicable diseases [5,6,7], and decreased quality of life [8,9]. Depression represents a major public health problem [10]; it is estimated that more than 300 million people suffer from depression worldwide, 4.4% of the population [2]. In Spain, depression is the most common mental disorder, with a prevalence of 5.5% in men and 7–8% of women in the 55–79 age group [11]. The main risk factors for depression include poverty, unemployment, life events (e.g., death of a loved one), physical illness, and substance use [2]. This disease causes high costs in public health systems: EUR 118 billion in Europe (61% from indirect costs due to sick leaves) and EUR 5005 million per year in Spain [12].

Physical activity (PA) is defined as any bodily movement produced by skeletal muscles that results in energy expenditure [13]. As our study focuses only on physical activity definition, the following PA indicators will be followed: Physical Activity Level (PAL) and Physical Activity Index (PAI). The PA’s beneficial effects on mental health have been widely demonstrated [14], including the improvement of depressive and anxious symptomatology [15], self-esteem and mood [16], physical self-perception [15], cognitive functioning [17], health-related quality of life [18], and stress prevention and reduction [19]. Moreover, physically active individuals report lower levels of depression than their physically inactive peers [20,21,22]. PA protects against the onset of depression regardless of age and geographical region [23,24]. It is widely accepted that mood states are highly dependent on endorphin secretion [25]. In this sense, PA increases the secretion of this hormone which reduces anxiety and depression levels [26], so the PA can be considered an antidepressant treatment [22]. In addition, PA appears to be as effective as psychotherapy for the treatment of mild to moderate depressive symptoms [14]. Different PA exercises, such as aerobic and strength/flexibility training, seem equally effective for treating depression and anxiety symptoms [14]. However, it is important to remember that depression is an illness, so specialised monitoring of depression remains essential. Although PA is an excellent complement that optimises results and recovery, it should never be taken as a treatment in isolation, as depression requires therapy and, in some cases, medication [14].

Mental health has gained attention during the COVID-19 pandemic, as increased anxiety and psychological distress episodes in the general population have occurred [27]. During the COVID-19 pandemic, positive associations between higher PAL and physical health, and inverse associations between sedentary behaviours and physical and mental health outcomes have been reported [28,29]. Moreover, a study reported that lack of social support was significantly associated with an elevated risk of depression and poor sleep quality [30]. In this regard, perceived social support (PSS) refers to the relationships that the individual establishes with their social network and the individual’s subjective evaluation of the adequacy of the received support [31,32]. PSS can be defined as “an exchange of resources between at least two individuals perceived by the provider or the recipient to be intended to enhance the well-being of the recipient” [33]. PSS has been more determinant than social support in mental health during the COVID-19 pandemic [34]. Therefore, PSS should be considered a significant predictor of well-being as it corresponds to the individual’s subjective assessment of their resources [35] and as a psychosocial protective factor concerning mental health. Several studies [28,29,30] suggested that proper PSS reduces anxiety levels and depression symptoms. Furthermore, adolescents’ PAL was associated with PSS and had a significantly positive effect on older adults’ PA [36,37].

Therefore, this study aims to explore the differences in PAL between male and female and analyse the associations between mental health and its dimensions assessed through the Goldberg’s General Health Questionnaire (GHQ-12) with the PAL and the PSS, measured by the Duke-UNC-11 Functional Social Support Questionnaire in the Spanish adult population with psychological distress. It was hypothesised that both a higher PAL and PSS would be related to lower indicators of psychological distress, according to the GHQ-12.

## 2. Materials and Methods

### 2.1. Design

This research is a cross-sectional study based on the Spanish National Health Survey 2017 (ENSE 2017) adult questionnaire [38], the last health survey conducted in Spain before the COVID-19 pandemic. The ENSE is a survey conducted in Spain every five years, promoted by the Ministry of Health, Consumer Affairs and Social Welfare (MSCBS) in collaboration with the National Statistics Institute (INE). It collects information on the health status of the population residing in Spain. For the ENSE 2017, trained and accredited interviewers conducted the surveys between October 2016 and October 2017.

### 2.2. Participants

A total of 23,089 people aged between 15 and 103 years, residents in Spain, were interviewed in the ENSE 2017. Participants were selected using a stratified three-phase random sampling system [39].

#### 2.2.1. Exclusion Criteria

Participants over 70 years of age were excluded as they were not questioned about PA (5312 participants). All participants who reported not suffering from depression were excluded (in the ENSE 2017, questions about depression included the items 25.20a (Have you ever been affected by depression?) and 25.20b (Has a doctor told you that you have it?). Participants who answered “No” to both questions were excluded (16,073)). Participants who did not answer any of the GHQ-12 items (47.1–47.12) were also discarded (34 participants). Finally, for analyses that included the Duke-UNC-11, participants with no responses on any of its items (130.1–130.11) were discarded (63 participants).

#### 2.2.2. Inclusion Criteria

The final sample consisted of 1670 individuals (525 men and 1145 women), aged between 15–69 years old, who reported suffering from depression (affirmative response to items 25.20a or 25.20b) and who responded to all items on the PA (113–117) and the GHQ-12 items (47.1–47.12); except to perceived social support where there were only 1607 valid answers (494 from men and 1113 from women).

However, our study does not focus on any kind of depression, but on the psychological distress of the participants. This is due to the fact that although the participant is asked if he/she is diagnosed with depression by a doctor, it is not possible to state this without verification by a psychiatrist.

### 2.3. Variables

**Mental health:** constructed from answers to items 47.1–47.12 from the ENSE 2017, those corresponding to the Spanish version of the Goldberg General Health Questionnaire (GHQ-12) [40], a multidimensional scale for mental health assessment. The GHQ-12 is a self-report tool used as screening to identify psychological distress and short-term changes in mental health, with high internal consistency (α = 0.86) in the Spanish population. The GHQ-12 values range from 0 to 36, as the items’ responses range from 0 to 3, conforming to an overall index with the sum of all responses. Thus, 0 represents the best mental health and 36, the worst. People with scores above 12 are considered to have some form of psychological distress [40,41,42,43]. The GHQ-12 is conformed to three dimensions [41,42]:

**Successful coping (FI)** was constructed with the sum of the responses to the items Q.47.1, Q.47.3, Q.47.4, Q.47.7, Q.47.8, and Q.47.12. Its scores range from 0 to 18, with 0 being the most successful coping and 18 being the least. This factor has an external validity of 0.82 with a *p*-value of 0.001 [41].

**Self-esteem (FII)** was constructed with the sum of the responses to the items Q.47.6, Q.47.9, Q.47.10, and Q.47.11. Its scores range from 0 to 12, with 0 being the highest self-esteem and 12, the lowest. This factor has an external validity of 0.70 with a *p*-value of 0.001 [41].

**Stress (FIII)** was constructed with the sum of the responses to items Q.47.2, Q.47.5, and Q.47.9. Its scores range from 0 to 9, with 0 being the least stressful and 9 being the most stressful. This factor has an external validity of 0.75, with a *p*-value of 0.001 [41].

**Perceived social support (PSS):** constructed with the responses to items 130.1–130.11, corresponding to the Duke-UNC-11 Functional Social Support Questionnaire, a questionnaire used to assess participants’ PSS. It consists of 11 items, with five possible responses, which can take values from 0 (much less than I want) to 5 (as much as I want). Thus, PSS is constructed with the sum of all the responses with values between 11 and 55. In the Spanish population, values below 32 indicate low PSS. This questionnaire’s internal consistency in this population is excellent (α = 0.90) [41,44,45].

**Physical Activity Index (PAI)** was used to assess the participants’ PA. This variable was created using the answers given in the ENSE 2017 to items P.113-P.116, corresponding to the Spanish version of The International Physical Activity Questionnaire (IPAQ) [46], for participants who performed moderate and intense PA. An adaptation of the PAI [47] was carried out using the formula: (intensity factor for vigorous activity x frequency factor for vigorous activity x duration factor for vigorous activity) + (intensity factor for moderate activity x frequency factor for moderate activity x duration factor for moderate activity). The PAI could take values between 0 and 67.5, with 67.5 being the highest value for PA [48].

**Physical Activity Level (PAL):** using the PAI together with the answers given to the item Q.117 (Now think about how much time you spent walking in the last 7 days), with possible answers: (“No day more than 10 min at a time”, or from 1 to 7 days), four PAL groups were created:

- Inactives: PAI = 0; reported not walking any day of the week for more than 10 min at a time.
- Walkers: PAI = 0; reported walking at least one day a week for more than 10 min at a time.
- Actives: presented a PAI between 1 and 30.
- Very actives: presented a PAI higher than 30.

### 2.4. Statistical Analysis

The SPSS statistical software version 25 (IBM SPSS, Chicago, IL, USA) was used to process the data. The level of significance assumed was 0.05. First, a Kolmogorov–Smirnov test was used to analyse data distribution; insufficient evidence assumed that variables followed a normal distribution. Therefore, continuous variables (age, PAI, mental health, successful coping, self-esteem, stress, and PSS) were presented with median and interquartile ranges, complemented with mean and standard deviation. The ordinal variable, the PAL, was presented with absolute frequency and relative frequency data. Non-parametric statistical tests were carried out: Mann–Whitney’s U to analyse differences between sexes in the continuous variables; chi-square statistic to examine the independence of sex and PAL; and the Kruskal–Wallis test to check possible differences at baseline between the medians of mental health and its dimensions in the different PAL groups, in the general population, and the subgroups by sex. Finally, Spearman’s correlation coefficients were calculated using the Bonferroni correction. Correlation values were interpreted following Cohen’s classification thresholds [49] 0.1 to 0.29, weak; 0.30 to 0.59, moderate; 0.60 to 0.79, high; ≥0.80, excellent.

## 3. Results

Table 1 shows the descriptive analysis of the sample showing differences in the median PA performed by men and women (*p* < 0.001) and the association between sex and PAL (*p* < 0.001). The general population on the GHQ-12 questionnaire showed a median of 15 points (men: 16. women: 15) with no significant differences between the two scores. PSS was 46 in the general population, higher in women than in men (47 vs. 45. *p* < 0.038).

Table 2 shows the scores obtained in the GHQ-12 questionnaire and its dimensions, according to the participants’ PAL. In the GHQ-12, the highest score was found in the Inactive group (19), with a higher score in inactive men than inactive women (20 vs. 18). The lowest scores were found in the very active groups, both in the general population (13) and males (12; females, 14). Differences were found in the baseline medians in the general population and both sexes according to the PAL (*p* < 0.001), as well as in the GHQ-12 dimensions.

Weak inverse correlations were found between PAL and mental health scores (rho: −0.214. *p* < 0.001), as well as with its three dimensions: successful coping (rho: −0.216. *p* < 0.001), self-esteem (rho: −0.209. *p* < 0.001), and stress (rho: −0.130. *p* < 0.001) according to the GHQ-12. Similarly, weak correlations were found between PAL and each of the GHQ-12 dimensions, resulting in statistically significant values (Table 3).

Finally, weak inverse correlations were found between the PSS and mental health scores (r: −0.307. *p* < 0.001) as well as with its three dimensions: Successful coping (r: −0.288. *p* < 0.001), Self-esteem (r: −0.302. *p* < 0.001) and Stress (r: −0.242. *p* < 0.001); according to the GHQ-12. Weak statistically significant inverse correlations between PSS and the GHQ-12 items were also found (Table 4).

## 4. Discussion

### 4.1. Main Findings

This study aimed to analyse the associations between mental health and its dimensions using the Goldberg’s General Health Questionnaire (GHQ-12), the PAL and PSS, using the Duke-UNC-11 Functional Social Support Questionnaire in the Spanish adult population with psychological distress. The main finding of this research was that higher PAL and PSS were correlated with better mental health in people with psychological distress.

Concerning PAL, significant associations between sexes, specifically in the average amount of PA performed by men and women, were found. The results highlighted that men performed more PA than women (8.4 vs. 5.1), in line with previous studies [50,51,52,53]. However, many more men were inactive (22.7% vs. 21.3%) compared to the Dumith et al. work [51]. At the same time, data indicated a higher percentage of physically active (20.6% vs. 17.7%) and very active (8.6% vs. 3.8%) men than women, in line with other studies [54,55]. Moreover, significant sex differences were found in PSS, being slightly higher in women (47 vs. 45. *p* < 0.038), as found in previous studies [56,57].

Regarding the GHQ-12, all the participants presented a score >12, considered the cut-off point for mental disorder occurrence [40]. Therefore, the results support the diagnosis of psychological distress in the participants sampled in this study. A weak inverse correlation was found between PAL and total mental health score, similar to previous works [58,59]. These findings suggest that individuals with higher PAL could have lower scores on the GHQ-12, which mean less psychological distress. The highest GHQ-12 scores in the inactive group were higher in men than in women (20 vs. 18). In other words, the most inactive population presented the greatest psychological distress [20,21,22], with a greater prevalence in males [60]. However, active women did not benefit from PA as men reduced psychological distress, in line with the evidence found in other research [61]. However, the lowest GHQ-12 scores were found in the most active groups, in the general population and the sexes subgroups, as reported in other studies [20,21,22].

Furthermore, this inverse correlation keeps in the three dimensions established in the mental health questionnaire: successful coping, self-esteem, and stress; as well as in each of the items of the GHQ-12. The PA’s most significant benefits were found in successful coping and self-esteem considering the questionnaire dimensions. At the same time, stress did not vary significantly, as no direct associations were found between the PAL and the stress dimension in men, as already reported by Allison et al. [59]. Therefore, several studies state that PA decreases and psychological distress from depression associations exist [15], as well as the dimensions of successful coping [62] and self-esteem [16], both in the general population and in both sexes.

Moreover, a weak inverse correlation was found between mental health scores and PSS, as in the study by Abril [63], which reports an indirect relationship between perceived social support and depression. Thus, this correlation retains the three dimensions: successful coping, self-esteem, and stress (as in the above-mentioned study by Abril [63]), as well as in the Duke-UNC-11 items. Our findings report that greater PSS is related to less psychological distress in people suffering from depression, as reflected in numerous studies [63,64,65]. In the study by Latkin et al. [64], the relationship between measures of social support and social integration and the level of depressive symptoms was examined, with results similar to ours. However, the data suggest that social support has only a minimal impact on reducing depression in impoverished settings. This may be due to the different measurement instruments used in the two studies (Center for Epidemiological Studies Depression Scale (CES-D) versus the Duke-UNC-11). In addition, the rest of the measures in this study can be considered subjective, as they come from an initial interview. Finally, the study by Liu et al. [65] sought to identify the association between depression and anxiety with the period of social isolation during COVID-19, obtaining results in line with those obtained in our research. Participants with higher social support were associated with lower levels of depression. In this case, they considered psychological resilience (CD-RISC-10), loneliness (short form of the UCLA Loneliness), and perceived social support, which was measured by the Multidimensional Scale of Perceived Social Support. In this sense, considering the similarity of the studies, it would be interesting to consider these new variables mentioned by others as well as measurement instruments for future studies.

### 4.2. Practical Applications

Reducing the psychological distress of people with depression could help reduce the health care costs and social losses associated with this disorder, especially given that this syndrome affects mainly low-income populations. Higher PAL and PSS are related to lower scores on the GHQ-12, which could imply less psychological distress in people with depression by increasing successful coping and self-esteem in this population and a stress reduction. Therefore, PAL and PSS could be considered essential predictors of well-being, positioning themselves as psychosocial protective factors related to mental health difficulties.

It would be advisable to implement physical exercise programmes that include moderate and intense walking and physical exercise several days a week as part of a multidisciplinary approach to treating people with depression. Combining physical exercise programmes with group activities, follow-ups, interaction points, or other alternatives could be interesting. At the same time, increasing the individuals’ PAL could reinforce their PSS, helping to minimise the mental distress caused by depression. As numerous studies have shown, PA reduces anxiety and depressive symptoms [24,66,67,68,69].

### 4.3. Limitations

This study has several limitations. Despite finding dependency relationships and correlations, causal relationships cannot be established, given the study design. Discovering the mechanisms by which PAL increases in people with depression should be a future line to follow. It has also been found that walking or moderate and intense PA could reduce GHQ-12 scores. It would be convenient to conduct research to establish optimal exercises doses. Moreover, the PA was not obtained through objective measures, such as inertial devices, and it would be of particular interest to incorporate this technology in future research. Another limitation was that different PAL among those who only walked was not created, not discriminating between those who walked more or fewer days or the frequency of moderate and vigorous PA, which could help to better understand the correlations found. Finally, it should be also noted that depression was not evaluated according to established criteria for it but was self-reported by participants since the study does not focus on any kind of depression specifically, but on the psychological distress.

## 5. Conclusions

Based on the presented results, we conclude that mental health and its dimensions are associated with PAL and PSS in the Spanish adult population with psychological distress. Specifically, people with higher PAL and PSS present lower psychological distress, according to the GHQ-12. Therefore, future studies should consider the analysis of the effects of physical activity interventions as a complement resource or strategy for psychological distress treatment.

## Figures and Tables

**Table 1:** Descriptive analysis includes age, physical activity index (PAI), the Goldberg General Health Questionnaire dimensions (GHQ-12), the Duke-UNC-11 Functional Social Support Questionnaire, and the PAL in Spanish adults with psychological distress, according to the ENSE 2017.

Variables	Total *n* = 1670	Men *n* = 525	Women *n* = 1145	*p*
Age (Years)				0.003 a
Median (IQR)	55 (17)	53 (16)	56 (17)	
Mean (SD)	52.8 (11.7)	51.7 (11.8)	53.3 (11.6)	
PAI				<0.001 a
Median (IQR)	0 (0)	0 (15)	0 (0)	
Mean (SD)	6.1 (13.1)	8.4 (15.4)	5.1 (11.7)	
Mental health				0.077 a
Median (IQR)	15 (10)	16 (11)	15 (10)	
Mean (SD)	16.4 (7.0)	17.0 (7.4)	16.2 (6.8)	
Successful coping				0.011 a
Median (IQR)	7 (4)	7 (5)	7 (4)	
Mean (SD)	8.2 (3.0)	8.5 (3.2)	8.0 (3.0)	
Self-esteem				0.149 a
Median (IQR)	4 (4)	5 (5)	4 (4)	
Mean (SD)	5.0 (3.2)	5.2 (3.3)	4.9 (3.1)	
Stress				0.474 a
Median (IQR)	5 (3)	5 (4)	5 (3)	
Mean (SD)	4.9 (2.3)	4.9 (2.3)	4.8 (2.2)	
Perceived social support	Total *n* = 1607	Men *n* = 494	Women *n* = 1.113	0.038 a
Median (IQR)	46 (13)	45 (13)	47 (13)	
Mean (SD)	44.0 (9.5)	43.2 (10.0)	44.4 (9.3)	
PAL				<0.001 b
Inactives	363 (21.7%)	119 (22.7%)	244 (21.3%)	
Walkers	907 (54.3%)	253 (48.2%)	654 (57.1%)	
Actives	311 (18.6%)	108 (20.6%)	203 (17.7%)	
Very actives	89 (5.3%)	45 (8.6%)	44 (3.8%)	

*n* (participants); % (percentage); IQR (interquartile range); SD (standard deviation); GHQ-12 (Goldberg’s General Health Questionnaire. Scores between 0 and 36: 0, the best mental health; 36, the worst mental health); Successful Coping (scores from 0 to 18: 0, the best coping; 18, the worst coping); Self-esteem (scores 0 to 9: 0, the best self-esteem; 9, the worst self-esteem); Stress (scores 0 to 9: 0, no stress; 9, very stressed); PAL (physical activity level); Inactive (PAI = 0; claim to not go for a walk, any day of the week, for more than 10 min at a time). Walkers (PAI = 0; report walking at least one day a week, more than 10 min at a time). Actives (PAI = 1–30); Very actives (PAI = +30); PAI (Physical Activity Index: scores between 0 and 67.5); a (*p*-value from U-Mann–Whitney test); b (*p*-value from chi-square test).

**Table 2:** Associations between the Goldberg General Health Questionnaire dimensions (GHQ-12), and PAL in Spanish adults with psychological distress, according to the ENSE 2017.

Variables	Total *n* = 1670			Men *n* = 525			Women *n* = 1145		
Mental health									
PAL	m (sd)	med (IQR)	*p*	m (sd)	med (IQR)	*p*	m (sd)	med (IQR)	*p*
Inactives	19.4 (7.4)	19 (11)	<0.001	20.3 (8.0)	20 (13)	<0.001	18.9 (7.0)	18 (10)	<0.001
Walkers	15.9 (6.9)	14 (10)		16.4 (6.8)	15 (11)		15.8 (6.9)	14 (9)	
Actives	15.0 (6.4)	14 (9)		15.8 (7.2)	15 (11)		14.6 (6.0)	14 (9)	
Very actives	13.6 (4.9)	13 (6)		12.8 (4.8)	12 (4)		14.3 (5.0)	14 (6)	
**Successful coping**									
PAL	m (sd)	med (IQR)	*p*	m (sd)	med (IQR)	*p*	m (sd)	med (IQR)	*p*
Inactives	9.5 (3.3)	9 (6)	<0.001	9.9 (3.7)	10 (7)	<0.001	9.2 (3.1)	9 (5)	<0.001
Walkers	8.0 (3.0)	7 (4)		8.2 (2.9)	7 (4)		7.9 (3.0)	7 (3)	
Actives	7.5 (2.6)	7 (3)		7.9 (2.8)	8 (4)		7.2 (2.5)	7 (2)	
Very actives	6.8 (1.9)	6 (2)		6.7 (1.9)	6 (1)		6.9 (1.9)	7 (2)	
**Self-esteem**									
PAL	m (sd)	med (IQR)	*p*	m (sd)	med (IQR)	*p*	m (sd)	med (IQR)	*p*
Inactives	6.2 (3.2)	6 (5)	<0.001	6.6 (3.4)	7 (5)	<0.001	6.0 (3.2)	6 (4)	<0.001
Walkers	4.7 (3.1)	4 (5)		4.9 (3.1)	4 (4)		4.7 (3.0)	4 (5)	
Actives	4.4 (3.1)	4 (5)		4.6 (3.4)	4 (5)		4.2 (2.9)	4 (4)	
Very actives	3.7 (2.4)	4 (3)		3.5 (2.4)	3 (2)		4.0 (2.4)	4 (3)	
**Stress**									
PAL	m (sd)	med (IQR)	*p*	m (sd)	med (IQR)	*p*	m (sd)	med (IQR)	*p*
Inactives	5.5 (2.2)	6 (3)	<0.001	5.5 (2.4)	6 (4)	0.001	5.5 (2.1)	6 (3)	<0.001
Walkers	4.7 (5.1)	5 (3)		4.9 (2.1)	5 (3)		4.7 (2.3)	5 (3)	
Actives	4.6 (2.3)	5 (3)		4.7 (2.5)	5 (3)		4.6 (2.2)	5 (3)	
Very actives	4.4 (2.2)	4 (3)		3.9 (2.1)	3 (3)		5.0 (2.1)	5 (3)	

m (mean); sd (standard deviation); med (median); IQR (interquartile range); GHQ-12 (Goldberg’s General Health Questionnaire. Scores between 0 and 36: 0, the best mental health; 36, the worst mental health); Successful Coping (scores from 0 to 18: 0, the best coping; 18, the worst coping); Self-esteem (scores 0 to 9: 0, the best self-esteem; 9, the worst self-esteem); Stress (scores 0 to 9: 0, no stress; 9, very stressed); PAL (Physical Activity Level: Inactive (PAI = 0; claim to not go for a walk, on any day of the week, for more than 10 min at a time). Walkers (PAI = 0; report walking at least one day a week, more than 10 min at a time). Actives (PAI = 1–30); Very actives (PAI = +30); PAI (Physical Activity Index: scores between 0 and 67.5); *p* (*p*-value from Kruskal–Wallis’ test).

**Table 3:** Correlations between PAL and the Goldberg General Health Questionnaire (GHQ-12) dimensions in Spanish adults with psychological distress, according to the ENSE 2017.

Target Variable	Overall		Men		Women	
	Rho	*p*	Rho	*p*	Rho	*p*
Mental Health	−0.214	<0.001	−0.254	<0.001	−0.198	<0.001
Successful Coping	−0.216	<0.001	−0.228	<0.001	−0.219	<0.001
Self-esteem	−0.209	<0.001	−0.253	<0.001	−0.189	<0.001
Stress	−0.130	<0.001	−0.174	<0.001	−0.110	<0.001
1. Have you been able to concentrate well on what you were doing?	−0.146	<0.001	−0.139	0.001	−0.153	<0.001
2. Have your worries caused you to lose sleep?	−0.074	0.002	−0.127	0.003	−0.050	0.002
3. Did you feel that you were playing a useful role in life?	−0.179	<0.001	−0.182	<0.001	−0.189	<0.001
4. Did you feel able to make decisions?	−0.190	<0.001	−0.222	<0.001	−0.179	<0.001
5. Have you felt constantly overwhelmed and under stress?	−0.131	<0.001	−0.168	<0.001	−0.114	<0.001
6. Have you had the feeling that you cannot overcome your difficulties?	−0.168	<0.001	−0.195	<0.001	−0.155	<0.001
7. Have you been able to enjoy your normal daily activities?	−0.191	<0.001	−0.242	<0.001	−0.170	<0.001
8. Have you been able to cope adequately with your problems?	−0.211	<0.001	−0.235	<0.001	−0.203	<0.001
9. Have you felt unhappy or depressed?	−0.134	<0.001	−0.167	<0.001	−0.120	<0.001
10. Have you lost confidence in yourself?	−0.195	<0.001	−0.246	<0.001	−0.174	<0.001
11. Have you thought of yourself as a worthless person?	−0.219	<0.001	−0.264	<0.001	−0.201	<0.001
12. Do you feel reasonably happy considering all the circumstances?	−0.148	<0.001	−0.154	<0.001	−0.148	<0.001

GHQ-12 (Goldberg’s General Health Questionnaire. Scores between 0 and 36: 0, the best mental health; 36, the worst mental health); Successful Coping (scores from 0 to 18: 0, the best coping. 18, the worst coping); Self-esteem (scores 0 to 9: 0, the best self-esteem, 9, the worst self-esteem); Stress (scores 0 to 9: 0, no stress, 9, very stressed); PAL (Physical Activity Level: Inactive (PAI = 0; claim to not go for a walk, on any day of the week, for more than 10 min at a time). Walkers (PAI = 0; report walking, at least one day a week, more than 10 min at a time). Actives (PAI = 1–30); Very actives (PAI = +30); PAI (Physical Activity Index: Scores between 0 and 67.5); Rho (Spearman’s correlation coefficients with the Bonferroni correction factor having *p* = 0.003); *p* (*p*-value).

**Table 4:** Correlations between perceived social support in the Duke-UNC-11 Functional Social Support Questionnaire and the Goldberg General Health Questionnaire (GHQ-12) in Spanish adults with psychological distress, according to the ENSE 2017.

Target Variable	Overall		Men		Women	
	Rho	*p*	Rho	*p*	Rho	*p*
Mental Health	−0.307	<0.001	−0.265	<0.001	−0.325	<0.001
Successful Coping	−0.288	<0.001	−0.252	<0.001	−0.302	<0.001
Self-esteem	−0.302	<0.001	−0.261	<0.001	−0.320	<0.001
Stress	−0.242	<0.001	−0.209	<0.001	−0.259	<0.001
1. Have you been able to concentrate well on what you were doing?	−0.160	<0.001	−0.136	0.003	−0.175	<0.001
2. Have your worries caused you to lose sleep?	−0.155	<0.001	−0.129	0.004	−0.167	<0.001
3. Did you feel that you were playing a useful role in life?	−0.171	<0.001	−0.195	<0.001	−0.153	<0.001
4. Did you feel able to make decisions?	−0.179	<0.001	−0.206	<0.001	−0.180	<0.001
5. Have you felt constantly overwhelmed and under stress?	−0.191	<0.001	−0.203	<0.001	−0.196	<0.001
6. Have you had the feeling that you cannot overcome your difficulties?	−0.211	<0.001	−0.217	<0.001	−0.227	<0.001
7. Have you been able to enjoy your normal daily activities?	−0.214	<0.001	−0.170	<0.001	−0.235	<0.001
8. Have you been able to cope adequately with your problems?	−0.200	<0.001	−0.205	<0.001	−0.209	<0.001
9. Have you felt unhappy or depressed?	−0.217	<0.001	−0.214	<0.001	−0.240	<0.001
10. Have you lost confidence in yourself?	−0.226	<0.001	−0.216	<0.001	−0.242	<0.001
11. Have you thought of yourself as a worthless person?	−0.217	<0.001	−0.255	<0.001	−0.212	<0.001
12. Do you feel reasonably happy considering all the circumstances?	−0.228	<0.001	−0.259	<0.001	−0.222	<0.001

GHQ-12 (Goldberg’s General Health Questionnaire. Scores between 0 and 36. 0, the best mental health. 36, the worst mental health); Successful Coping (Scores from 0 to 18. 0, the best coping. 18, the worst coping); Self-esteem (Scores 0 to 9. 0, the best self-esteem. 9, the worst self-esteem); Stress (Scores 0 to 9. 0, no stress. 9, very stressed); Duke-UNC-11 (Duke-UNC-11 Functional Social Support Questionnaire. Scores between 11–55 points); Rho (Spearman’s correlation coefficients with the Bonferroni correction factor having *p* = 0.003); *p* (*p*-value).

## Data Availability

Data used were obtained from public use files, available on the Spanish Ministry of Health, Consumer Affairs, and Social Welfare website: https://www.mscbs.gob.es/estadEstudios/estadisticas/encuestaNacional/encuesta2017.htm (accessed on 1 June 2022). Additional datasets will be available upon reasonable request.
